# Lipids, Lipoprotein Distribution and Nutritional Parameters over the Ramadan Period in Hemodialysis Patients

**DOI:** 10.3390/nu11092225

**Published:** 2019-09-14

**Authors:** Bayan Tashkandi, Deepinder Kaur, Eno Latifi, Dina A. Tallman, Karuthan Chinna, Zulfitri Azuan Mat Daud, Tilakavati Karupaiah, Hanadi Alhozali, Pramod Khosla

**Affiliations:** 1 Department of Nutrition and Food Science, Wayne State University, Detroit, MI 48202, USA; bayan.tashkandi@wayne.edu (B.T.); kdeepinder@wayne.edu (D.K.); eno.latifi@wayne.edu (E.L.); dina.tallman@wayne.edu (D.A.T.); 2 School of Medicine, Faculty of Health and Medical Sciences, Taylor’s University, Subang Jaya 47500, Malaysia; karuthan@gmail.com (K.C.); tilly_karu@yahoo.co.uk (T.K.); 3 Department of Nutrition and Dietetics, Faculty of Medicine and Health Sciences, University Putra Malaysia, 43400 UPM Serdang, Malaysia; zulfitri@upm.edu.my; 4 Department of Nephrology, KAU Hospital, King Abdulaziz University, Jeddah 21589 P.O box 80215, Saudi Arabia; hanadi.alhozali@gmail.com

**Keywords:** Ramadan, hemodialysis, plasma lipids, lipoprotein particles, nutrition, anthropometrics

## Abstract

The period of Ramadan (R) is associated with dramatic changes in eating habits involving extended periods of fasting on a daily basis. The current study assessed whether lipids and lipoproteins were impacted during R in chronic hemodialysis (HD) patients. Forty-five subjects in an outpatient dialysis clinic in Saudi Arabia were evaluated for anthropometric and lipid changes on a monthly basis before, during as well as one and two months after R. In addition to routine biochemical measures, anthropometric assessments including hand-grip strength (HGS), mid-arm muscle circumference (MAMC), plasma lipids and lipoproteins were evaluated. Dietary assessment was carried out using 24 h recalls. Over the course of the study, changes in renal-related parameters (creatinine, albumin, Kt/V) were minor, as were changes in plasma lipids. Large high-density lipoproteins (HDLs) and low-density lipoproteins (LDLs) accounted for the majority of their respective lipoproteins and their proportions did not change over the study period. Mean LDL particle diameters were higher during the R period, but the changes over the study period were small. Calorie intake during R (2139 ± 709 kcal/d) was significantly higher than the value noted two month post-R (1755 ± 424 kcal/d) and this was associated with significant increases in protein (69 ± 24 vs. 60 ± 24 g/d) and fat (97 ± 38, vs. 73 ± 35 g/d), respectively. No changes were noted with respect to HGS and MAMC. These data show that for HD patients, the period of R results in temporal or non-significant effects on plasma lipids, despite changes in nutrient intake.

## 1. Introduction

Healthy Muslims are required to fast annually during the month of Ramadan (R). Fasting, which lasts from dawn to dusk can vary between 12–16 h. However, sick people, travelers, nursing, pregnant or menstruating women are exempt from this act of faith. The lifestyle change made for fasting adherence can dramatically result in alterations in the type and amount of food consumed, resting metabolic rate as well as physical activity levels [1,2,3,4,5,6].

For patients with chronic kidney disease (CKD), the month of R can pose serious challenges. With progression of CKD, dietary restrictions become more prominent resulting in stricter limitations on the intake of specific nutrients. While CKD/ End Stage Renal Disease (ESRD) patients are advised against fasting, many choose to do so and in some instances, some opt to fast on non-dialysis days [7,8]. In a study of 65 patients with CKD (stages 3–5), Bakhit et al. [9] noted a worsening of kidney function during R in 33% of patients, based on increases in serum creatinine, but these levels normalized three months post-R (in a subset of patients). In a study of 15 CKD patients, El-Wakil et al., [10] while noting no changes in creatinine, found an impairment in renal tubular injury based on a urinary biomarker. Results across studies differ, especially in pre-dialysis patients as the degree of renal failure of the studied subjects is not the same.

In contrast, Bernieh et al. [8] evaluated 31 CKD patients (stages 3–5) and found no effects on creatinine or any other adverse changes during R [11]. In a study with 39 CKD patients (stages 2–5) no changes were noted in relevant clinical markers before, during or after R. Studies done in individuals with normalized renal function (e.g., post-transplant kidney patients or healthy individuals) have shown no adverse effects during R on several renal-related parameters [12,13,14,15,16,17,18].

With regards to CKD (stage 5) patients undergoing hemodialysis (HD), dietary restrictions can be pronounced. In the case of these individuals, the dialysis regimen itself requires additional coordination with the fasting schedule during R. Additionally, HD patients are at an increased risk for cardiovascular disease (CVD) partly attributed to dyslipidemia, increased inflammation, and poor nutritional status [19,20,21,22,23], and they could promote low-density lipoprotein (LDL) oxidation, endothelial injury, and the accumulation of lipids in the artery wall [24]. On the other hand, a diet approach could be beneficial to control them [24,25]. The findings from several studies in Saudi Arabia report that poor nutritional status and high prevalence of malnutrition (among HD patients) are consistent with these observations [23,26,27]. In HD patients, changes in various clinical and biochemical parameters during R have been reported in relatively few studies, and generally no adverse effects have been noted [7,8,28]. The impact or association of any nutritional changes with changes in anthropometric or blood chemistry (including lipids) is largely unexplored. Recently Adanan et al. [29] reported on nutritional and anthropometric changes in Malaysian subjects before, during and after R. Although temporal changes in some nutritional parameters were noted, none were considered detrimental.

In HD subjects with dyslipidemia (high plasma triacylglycerol (TAG) and low high-density lipoprotein cholesterol (HDL-C) concentrations), changes in dietary habits (as in the case during R), regardless of whether subjects opt to fast the entire stretch or fast for only a portion of the R period, may affect circulating lipoproteins. There is however limited information available on alterations in plasma lipids [30] during the period of R in HD patients. Wan Md Adnan et al. [30] noted decreased low-density lipoprotein cholesterol (LDL-C) concentrations at the end of R in diabetic HD patients, while Adanan et al. [29] reported no effects on LDL-C. Mixed results have also been obtained in healthy individuals where increased LDL-C and lower TAG [15], or no effects on LDL-C [31] or decreased LDL-C [29,32] have been observed during the R period. In contrast, a meta-analysis in healthy subjects during R found a significant reduction in LDL in both genders, a significant reduction in total cholesterol and TAG amongst males and a significant increase in HDL-C among females [16].

Since the dyslipidemia environment in HD patients is associated with perturbations in lipoprotein metabolism, we evaluated lipids and lipoproteins including LDL, HDL and their subfractions. In addition, we documented biochemical parameters as well as diet and anthropometric measures at monthly intervals pre-, during and one and two months post-R in HD patients. Finally, the potential role of diet in influencing lipoprotein composition was explored.

## 2. Methods

### 2.1. Study Design and Subjects

A prospective cohort study was conducted in an outpatient hemodialysis clinic at King Abdul Aziz University Hospital, Jeddah, Kingdom of Saudi Arabia in the summer of 2017 (Ramadan month comprised 29 days and dawn–dusk time period was ~10 h). Hemodialysis patients aged >18 years, not pregnant and those who had been on dialysis for at least 3 months prior to the study were enrolled. Informed written consent was obtained from all participants, and the study was approved by the Unit of Biomedical Ethics Research Committee at King Abdul Aziz University Ref #263-17.

**Figure 1:**
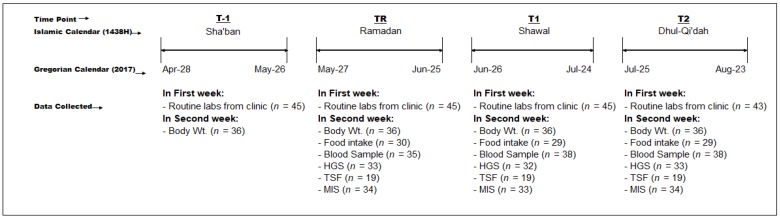
Study flow.

Data was collected monthly, (pre-Ramadan (T-1), during Ramadan (TR), 1 month post-Ramadan (T1]) and 2 months post-Ramadan (T2)), as indicated in Figure 1.

### 2.2. Sociodemographic and Health Data

Sociodemographic and clinical data were collected from medical records or by directly asking participants multiple choice or single item questions (yes or no) as detailed in Table 1. Additionally, monthly blood sampling was used to generate biochemical reports (Table 2 and Supplemental Table S1).

### 2.3. Anthropometric Assessment

Pre- and post-dialysis weight and height were obtained from the medical records to calculate body mass index (BMI), using Quetelet’s index (BMI = body weight (kg)/height (m^2^) [33]).

After the dialysis session, with subjects in a standing position, mid-arm circumference (MAC) was measured using a non-stretchable Lufkin^®^ tape (Apex Tool Group, LLC, NC, USA). Also, triceps skinfold thickness (TSF) measurements were obtained using a Harpenden skinfold caliper (HSK-BI, British Indicators, West Sussex, UK). All measurements were based on the International Society for Advancement of Kinanthropometry protocol. Mid-arm muscle circumference (MAMC) and corrected midarm muscle area (cAMA) were calculated based on published equations [34]. To assess muscle strength, handgrip strength (HGS) was measured by taking three readings from the non-fistula hand using a JAMAR dynamometer (Model # BK-7498; Fred Sammons, Inc., Burr Ridge, IL, USA) with a rest period of at least 1 min between trials. The measurements were taken post-dialysis while the participant was sitting (using the American Society of Hand Therapists standard protocol) [35]. The highest value was used in the analysis.

### 2.4. Nutritional Assessment

Food intake was evaluated using (i) one 24-h recall during Ramadan (TR), obtained on a non-dialysis day, and (ii) 24-h recalls obtained on 1 dialysis, 1 non-dialysis, and 1 weekend day at T1 and T2. Standard household measures were used to facilitate portion size recall by patients and all information related to diet was captured by the same researcher. Food intake and nutrients were analyzed using the Food Processor Program (version 11.2.274, ESHA Research, Salem, OR). For mixed dishes that were not in the Food Processor Program, data (name and amount) for each individual ingredient was entered in the recipe builder within the Food Processor Software to get the nutritional analysis, then the nutritional information (calories and macronutrients) was verified by searching various food database websites and comparing them.

To minimize systemic errors in dietary records, under-reporting of energy intake was evaluated by calculating the ratio between reported energy intake (EIrep) and total energy expenditure (TEE). Generally, overreporting is not common in HD patients, and in healthy individuals during R; higher calorie intake may actually be prevalent [4,36,37]. Adanan et al. [29] noted no over-reporting in food intake. We therefore analyzed data only for under-reporters, with a ratio of EIrep/TEE <0.76 being used as the cut-off for under-reporting of EIrep [36]. The TEE was estimated by calculating basal metabolic rate (BMR) using the Harris and Benedict equation [38] with an activity factor of 1.35 [39]. As we did not examine physical activity levels (PALs), the lowest PAL associated with weight maintenance (i.e., 1.35) was used. This factor represents habitual activity patterns in free-living individuals, assuming they are sedentary. This is also consistent with the Goldberg cut-off formula which uses a PAL of 1.35 to identify under-reporters of energy intake [39]. When their weight was <95% or >115% of the standard body weight we used adjusted edema-free body weight; while when the value was between 95%–115% of the standard body weight we used actual body weight as recommended by National Kidney Foundation/Kidney Disease Outcomes Quality Initiative (NKF KDOQI) (2000) guidelines [40].

### 2.5. Malnutrition-Inflammation Score

Malnutrition-inflammation score (MIS) questionnaire was collected from the participants during TR, T1 and T2. The MIS questionnaire included ten components, and each was scored between 0 (normal) to 3 (very severe) [41]. Thus, the sum of all the component scores ranged from 0 to 30. A higher total score indicates a more severe degree of malnutrition and inflammation.

### 2.6. Blood Sampling and Lipid Measurements

Monthly, pre-dialysis blood samples, collected into Ethylenediaminetetraacetic acid (EDTA) and lithium heparin-containing tubes, were obtained over the course of the study. Consistent with recent reports and guidelines (e.g., European Atherosclerosis Society and the European Federation of Clinical Chemistry and Laboratory Medicine) noting that the use of non-fasting blood samples for lipid analyses can be valid predictors of CVD risk, subjects were not required to provide fasting blood samples [42]. Samples were centrifuged on site at 3500 rpm for 10 min to separate plasma, and aliquots were stored at −80 °C.

Plasma samples were shipped frozen via courier (Aramex Delivery Unlimited, Dubai, UAE) to Wayne State University, 10 months later. Plasma total cholesterol (TC) and TAG were measured using enzymatic assays (Point Scientific Inc., Canton, MI, USA). HDL-C was measured in the supernatant after precipitating apo B-containing lipoproteins with dextran sulfate and magnesium ions (Point Scientific Inc.). LDL-C was calculated using the Friedwald formula (LDL − C = TC − HDL − C − (TAG/5)).

Plasma samples were also analyzed for HDL and LDL subfractions using the Lipoprint^TM^ (Quantimetrix Corporation, Redondo Beach, CA, USA) polyacrylamide electrophoresis-based system. The system separates lipoproteins based on size suing pre-cast gels. Following electrophoresis, LDL and HDL subfractions were quantitated using the manufacturer’s proprietary software. LDL is separated into 7 subfractions, which can then be classified into three groups constituting of large, intermediate and small LDL. Similarly, HDL can be separated into 10 subfractions which can be grouped into large, intermediate and small HDL. The Lipoprint^TM^ system is U.S. Food and Drug Administration (FDA) certified for LDL measurements, while values for HDL are for research purposes only.

### 2.7. Statistical Analyses

Data were analyzed using SPSS (Version 24, SPSS Inc., Chicago, Illinois, USA). Mean and standard deviation (SD) were calculated for continuous variables and categorical variables were described as frequencies and percentages. For the analyses an intention-to-treat (ITT) protocol was used, whereby missing values were imputed as the last known values. To study the changes over time, the generalized estimated equation procedure (GEE) was used. GEE is useful even if the correlation between the outcomes is not known. The GEE also allows for robust standard error or sandwich estimates. For all tests, the level of significance was set as *p* < 0.05.

## 3. Results

The demographics of the subjects are shown in Table 1. There were comparable numbers of males and females with a mean age of 50 years. Some 60% of the cohort identified themselves as non-Saudi and included nine different nationalities. Almost 70% of the subjects were married. Some 36% had a college education, while 31% had no formal education. Only 20% of the cohort was employed, while 47% of the cohort lived in homes with between three and five individuals, and some 27% lived in homes with more than five individuals. Almost 98% of the subjects were dialyzed thrice a week, with a mean dialysis session of 3.2 h, with a vintage of ~6.5 years. Further, 90% of the cohort were dialyzed by means of a catheter or fistula. The primary cause of renal failure was hypertension (49%) followed by diabetes (27%). In addition to diabetes (36%), secondary hyperthyroidism was diagnosed in 33% of the cohort. Only 11% of the cohort used tobacco.

The biochemical measures over the course of the study are shown in Table S1 and Table 2. In all, data for 12 parameters were available. There was no significant difference over the course of the study in Kt/V, calcium, ferritin or phosphorus. Transient increases were noted in random blood glucose (T1) and vitamin D levels (TR) (Table S1). In the case of albumin, K, total iron binding capacity (TIBC), pre- and post-blood urea nitrogen (BUN), creatinine and Na, variations were noted over the course of the study (Table 2). However, the range in values over the course of the study were: albumin 3.0–3.2 g/dL; 4.7–5.0 mEq/L; TIBC 181–235 mg/dL.

Table 3 shows the plasma lipid values during the course of the study (no samples were available for analyses from the T-1 time point). As compared to the values observed during Ramadan, the values obtained 2 months post-Ramadan (T2) were significantly higher for TC (184 ± 48 vs. 169 ± 55 mg/dL), HDL-C (40 ± 14 vs. 36 ± 10 mg/dL) and LDL-C (114 ± 39 vs. 104 ± 45 mg/dL) but were not significant for TAG (162 ± 55 vs. 143 ± 104 mg/dL). These changes were associated with significant differences in the values of TC/HDL-C and LDL-C/HDL-C when TR was compared with T2 values and there were no significant differences in the value of TAG/HDL-C.

**Figure 2:**
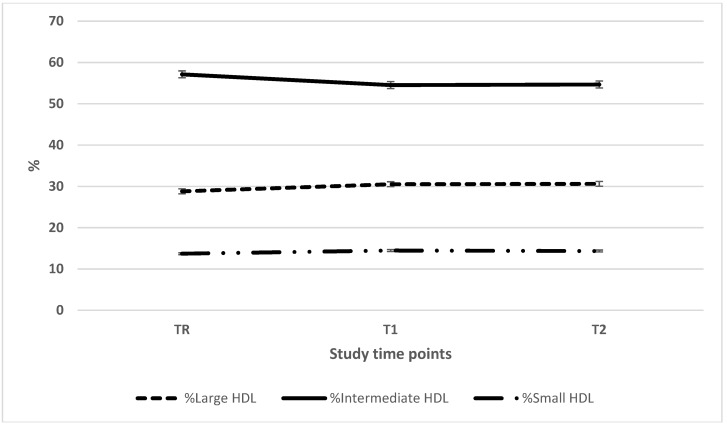
HDL particle size distributions. The percentage of HDL particle size (large, intermediate and small) distributions during the study period. Abbreviations: HDL: high-density lipoprotein; TR: during Ramadan; T1: one month post-Ramadan; T2: two months post-Ramadan. There is a significant difference for % intermediate HDL between TR and T1 and between TR and T2 at *p* < 0.5 level.

**Figure 3:**
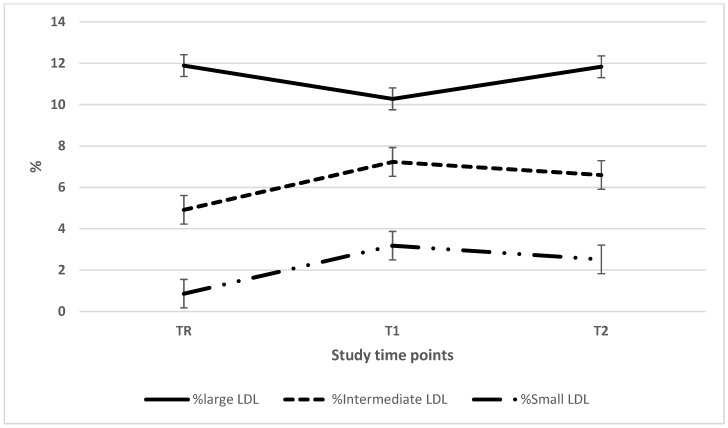
LDL particle size distributions. The percentage of LDL particle size (large, intermediate and small) distributions during the study period. Abbreviations: LDL: low-density lipoprotein; TR: during Ramadan; T1: one month post-Ramadan; T2: two months post-Ramadan. There is a significant difference for large LDL between TR and T1, and T2 and T1, also, for i- LDL and s-LDL between TR and T1 and between TR and T1 at *p* < 0.5 level.

To obtain further information on lipoprotein particle size distribution, plasma was electrophoresed using the Lipoprint system. As seen in Figure 2, based on the cholesterol distribution, intermediate-sized HDL (i-HDL) accounted for ~55% of the total HDL pool, followed by large-sized HDL (l-HDL) ~30% and small-sized HDL (s-HDL) ~14%. The HDL large and small particle size distribution was relatively consistent across time points; however, i-HDL was significant lower in TR when compared to T2. With regards to LDL (Figure 3), large-sized LDL (l-LDL) accounted for the bulk of the LDL particles, followed by intermediate-sized LDL (i-LDL) and small-sized LDL (s-LDL). There were significant shifts in particle distribution over the course of the study period. In terms of absolute values for cholesterol carried in the various HDL and LDL subfractions (Table 4), HDL values observed two months post-Ramadan were significantly higher than the values noted during Ramadan (l-HDL 13.8 ± 9.7 vs. 11.2 ± 7.1 mg/dL, i-HDL 21.3 ± 6.7 vs. 19.7 ± 4.4 mg/dL and s-HDL 4.9 ± 2.4 vs. 4.2 ± 2.0 mg/dL). Similarly, for cholesterol carried in LDL, the corresponding values were (i-LDL 13.6 ± 10.0 vs. 89.0 ± 8.4 mg/dL and s-LDL 5.3 ± 9.0 vs. 1.8 ± 3.0 mg/dL, respectively). There was no change for the corresponding values for l-LDL. LDL particle diameters that changed over the study period were significant when TR was compared with T2.

Table 5 shows the anthropometric assessment and malnutrition-inflammation score results. Values for BMI and pre- and post-dialysis weights did not change over the course of the study. No differences were noted in hand grip strength, MAMC and cAMA over the study period. Mean TSF values were significantly higher at T2 as compared to the values seen during Ramadan (21.1 ± 10.1 vs. 16.1 ± 17 mm). The MIS total score was significantly lower at T2 as compared to the values observed during Ramadan (10.0 ± 0.7 vs. 11.0 ± 0.7).

Table 6 details the nutrition and diet assessment over the course of the study. Both unadjusted and adjusted data are shown. The latter accounts for under-reporters. Total energy intake was significantly higher during Ramadan as compared to the values observed two months post-Ramadan in terms of total calories (2139 ± 709 vs. 1755 ± 424 kcal) as well as kcal/kg ideal body weight (33 ± 12 vs. 27 ± 5). The increased caloric intake reflected increased calories from protein (69 ± 24 vs. 60 ± 23, g/d *p* < 0.05) and fat (97 ± 38 vs. 73 ± 25, g/d *p* < 0.05), but not carbohydrates (248 ± 99 vs. 221 ± 70 g/d, *p* = Not Significant (NS)). Potassium intake was also significantly higher during Ramadan as compared to the values seen at T2 (1552 ± 846 vs. 1088 ± 406 mg/d, *p* < 0.05). Phosphate intake did not differ significantly during the study period.

## 4. Discussion

Studies conducted during R on HD patients are few and inconclusive. In this present study we evaluated biochemical parameters, food consumption and anthropometric measures, at monthly intervals pre-, during and 1 and 2 months post-R. Additionally, we evaluated lipids and lipoproteins including LDL and HDL subfractions during the study period. The most notable features to emerge from our analyses was constant body weight and muscle strength, high malnutrition-inflammation score, high total energy consumed (consistent with a significant intake of protein and fat), high HDL-C and significantly low levels in large HDL, as well as intermediate and small LDL, during R. However, most of these changes were transitory, consistent with recent findings [29].

We did not find any changes in body weight in our study and similar results have been obtained in other studies in HD patients [7,8,28]. In contrast, Wan Md Adnan et al. [30] found a significant reduction in body weight during R in HD patients. These differences in body weight could be because of the differences in food habits in different cultures as well as the fact that physical activity levels may vary especially due to outside temperature and climate. Additionally, temporal changes in energy intake may not always translate into measurable clinical changes in body weight. Malnutrition inflammation scores were significantly higher during R compared to T2, and this could be due to the change in lifestyle habits and quality of life. However, the MIS was low and in the ‘desirable’ range throughout the study. A study in fasting and non-fasting individuals during R found both groups modified their lifestyle during R [43]; also, Rambod et al. [44] found that MIS correlated with quality of life. Muscle strength did not change significantly in R compared to T2; similar results were noted by Albed et al. [45] in healthy individuals. In contrast, Adanan et al. [29] showed improvement in HGS. Muscle strength during the month of R among HD patients has not been thoroughly investigated; also, no reference values for muscle strength of HD patients are available [46]. Additionally, the period of R (30 days) may be too short to have any meaningful and sustained impact on muscle strength. Regarding biochemical assessment, the current study found serum albumin levels during R did not fluctuate appreciably over the study period. This result is in agreement with previous studies [8,28], while other studies have reported significant changes in serum albumin levels during R [7,29,30]. There may be several reasons for these differences including age of subjects, extent of renal impairment, study design, times of sampling as well as food habits. With reference to the latter, food habits across Pakistan [7], Saudi Arabia [8,28] and Malaysia [29,30] will vary considerably, regardless, our data say that across a three-month period encompassing R, albumin levels did not fluctuate appreciably to warrant any clinical concern.

Our study noted lower serum potassium during R consistent with a previous study [8]. However, other studies have noted no changes [7,28,30]. We noted increased total calorie consumption during R compared to T2 and this is in agreement with previous studies that were done in healthy participants from Saudi Arabia as well as from other countries [4,47,48]. This increase mainly came from both protein and fat. In contrast, some studies have shown a reduction in food intake [15,37]. However, in these studies the method of assessing diet intake as well the number of days for diet collection are different and this may have influenced the results. Additionally, the patterns and types of food intake are different across countries and cultures, and this could explain the effects on body weight observed in some. However, the stability of body weight in our study did not correlate with high caloric intake during Ramadan and this could be because we used only one 24-h diet recall during this month.

An additional reason for differences across studies may relate to the protocols employed and the times between sampling. Al Wakeel et al. [8] assessed measures one week before R, then between 7–15 days during R, and at the end of R. Imtiaz et al. [7] assessed parameters two weeks before R and during the last week of R; since there is a difference of six weeks in both of these studies, it is not possible to ascertain to what extent the changes observed during R were sustained once R ended. Adanan et al. [29] assessed measures two weeks before R, at the end of R and one month post-R. Thus, they were able to capture changes at a time post-R, that was equal to the period of R, and noted no differences. Our results are essentially in agreement with these observations [29], and since we had a uniform monthly sampling schedule (Figure 1) we were able to capture data one and two months post-R. However, even though we collected data every four weeks, the diet data captured was in the second week of R and second week of T1 and T2.

Overall, there was no significant change in TC, TAG and LDL-C over the course of the study. However, HDL-C levels increased significantly during R compared to T2. In contrast, Wan Md Adnan et al. found a significant reduction in HDL-C but only in diabetic patients who opted to fast during R [30]. We did not observe any consistent effects on particle size distribution during the course of the study. In studies examining intermittent fasting, which reflects intermittent energy restriction, increases in LDL particle sizes, not necessarily accompanied by changes in LDL-C, have been observed in obese subjects [49,50,51,52,53]. However, to the best of our knowledge, no comparative studies have assessed the effect of the R month on lipid subfractions in HD individuals and scant published values are available for us to compare.

## 5. Limitation of Our Study

Our study has several limitations. First, we were not able to capture a complete dataset for all patients at all time points. In the instance of missing lipid values at T-1, the values obtained at T2 may actually be more representative of the non-R period, given that lipoproteins generally stabilize within three to four weeks. Additionally, even though we collected data every four weeks, the collection was in the middle of the time period (T-1, TR, T1, T2). It is possible that the diet intake, if assessed other times during TR, may have been different.

Second, as with all studies involving R, there are variations in the length and frequency of the daily fast; some subjects may fast daily over the entire month, while others may fast 80%–100% of the time. Obtaining an exact measure on the latter is difficult to assess as it is a culturally sensitive topic. Individuals who do not participate in the fasting ritual either in part or for the entire month may be reluctant to divulge this information. In the case of dialysis patients, additional inconsistencies exist across the literature in terms of the protocols employed to obtain blood samples. To collect a fasting sample would necessitate blood collection in the evening. In some studies, some or all blood samples are taken at night consistent with a nightly dialysis schedule [8,29]. While we did not ask for fasting blood samples from our subjects, we do not believe this was a factor in our results. Collectively, 42.6% of all the measured TAG values in our samples were <100 mg/dL and 77.8% were <150 mg/dL. This suggests that TAG values were not necessarily post-prandial and most probably reflected values likely to be found during a fasting period. However, to truly ascertain whether subjects were fasting would necessitate measurements of apoB-48. In the recent study of Adanan et al. [29], subjects self-reported the number of days that they fasted during R. Based on that, subjects were divided into groups that fasted >20 days and those that fasted less than 20 days. In the case of the latter, this number varied between 4 and 19 days. Although no values for TAG were reported, LDL and HDL values were consistent with what we observed and did not change over the study period. It is of interest to note that amongst HD patients, the fact that subjects undergo blood exchange during the procedure is considered a “breaking of the fast” from a religious standpoint, by some scholars. It is also to be noted that recent guidelines from various agencies suggest that non-fasting lipids are indeed reliable measures for predicting CVD risk. In this regard three large cohort studies using data from a national dialysis provider were able to correlate lipid values (fasting indeterminate) with mortality rates [54,55,56].

Nutrition assessment during R is fraught with the additional problem that individuals who actually overreport may be difficult to separate from those who genuinely consume excess nutrients during the festival, although this is unlikely for dialysis patients who are generally on restrictive diets. Our sample size did not allow for determination of overreporters with any confidence. We did find 28% of our subjects under-reported (8/29), while the corresponding figure in the study by Adanan et al. [29] was 25% (21/83).

Finally, practices and food consumption during R vary across cultures and may result in shifts in nutrients unique to specific cultures. As an example, we noted increased protein and fat consumption but not carbohydrate intake which may have reflected that we had several nationalities within our cohort who would likely have had different food habits. This is in contrast to the recent report from Malaysia [30] where no change in energy intakes during R was noted.

Despite the above limitations, our study has several strengths. First, we employed a sampling schedule, separated by discreet one-month intervals that allowed us to capture data up to two months post-R. Second, to our knowledge, this is one of the first studies in HD patients to capture data on biochemical, anthropometric and plasma lipoproteins. The latter analyses were made more robust with our evaluation of different lipoprotein subfractions and particle sizes. In terms of overall health effects on HD patients over the course of our study, the data suggest that in addition to lipoproteins, renal-specific parameters were also relatively unchanged.

In conclusion, our data shows that lipoprotein fluctuations during R were temporary in our small sample of HD patients in Saudi Arabia. Whether similar trends would be observed in other ethnic/cultural groups needs to be established. A coordinated multi-country study with a standardized protocol for diet capture may be needed to address this question.

## Figures and Tables

**Table 1:** Demographic and clinical characteristic of subjects.

**Demographic Characteristics**	
Age (years)	50 ± 17
Gender (males, %)	22 (49%)
Nationality (Saudis, %)	18 (40%)
Marital Status	
Single/Married/Divorced	29%/69%/2%
Education	
Elementary/Middle/High schools	11%/11%/11%
College/None	35.5%/31.1%
Employed (Yes)	9 (20%)
No People in Home	
<3; 3–5; >5	27%/47%/27%
**Clinical Characteristics**	
Dialysis Session Duration (hours)	3.22 ± 0.12
Dialysis Frequency	
3 days/week	44/45 (97.8%)
Dialysis Vintage (months)	78 ± 61
Vascular Access	
Catheter/Fistula/Graft	40%/51%/9%
Cause	
DM/HTN/SLE/Others	27%/49%/7%/17%
Comorbidities	
DM/Sec. HPTH/Hep. C/Smokers	36%/33%/2%
Smokers	5 (11%)

Data are mean ± SD; *n* = 45 or percentages. Abbreviations: DM: diabetes mellitus; HTN: hypertension; SLE: systemic lupus erythematosus; Hep. C.: hepatitis C. Non-Saudi nationalities included: Yemeni 10, Filipino 5, Chadian 1, Palestinian 2, Eritrean 3, Myanmar 2, Somalian 1, Sudanese 2, and Syrian 1.

**Table 2:** Biochemical profiles.

Time	Pre-Ramadan (T-1)	Ramadan (TR)	1 month post -Ramadan (T1)	2 month post-Ramadan (T2)
Albumin (g/dL)	3.0 ± 0.38 (45)^ab^	3.2 ± 0.33 (45)^ac^	3.2 ± 0.40 (45)^bd^	3.1 ± 0.49 (43)^cd^
Potassium (mEq/L)	5.0 ± 1.5 (45)^a^	4.7 ± 0.91 (45)^b^	4.9 ± 90 (45)	5.0 ± 0.91 (43)^ab^
TIBC (mg/dL)	219 ± 58 (39)^a^	181 ±33 (40)^ab^	235 ± 72 (17)	211 ± 74 (42)^b^
BUN Pre-D (mg/dL)	47.5 ± 15 (45)^a^	51.9 ± 13 (45)	53.7 ± 13 (45)^a^	51.3 ± 15 (43)
BUN Post-D (mg/dL)	15.4 ± 6.3 (40)^abc^	18.0 ± 6.4 (41)^a^	18.8 ± 7.9 (41)^b^	18.9 ± 9.7 (41)^c^
Creatinine (mg/dL)	9.2 ± 3.0 (44)^abc^	9.9 ± 3.0 (44)^a^	10.5 ± 3.3 (44)^b^	10.1 ± 3.4 (42)^c^
Sodium (mEq/L)	135.0 ± 3 (45)^ab^	133.7 ± 3 (45)^ac^	136.1 ± 5 (45)	136.8 ± 3 (43)^bc^

Values are reported as mean ± SD for the numbers in parentheses. Abbreviations: Pre-D. Wt.: pre-dialysis weight; TIBC: total iron binding capacity; BUN pre-D: blood urea nitrogen pre-dialysis; BUN post-D: blood urea nitrogen post-dialysis. ^abcd^ Values in a given row with common superscripts were significant at *p* < 0.05. Creatinine 9.2–10.5 mg/dL, pre-dialysis BUN 47.5–53.7 mg/dL and post-dialysis BUN 15.4–18.9 mg/dL were within acceptable clinical guidelines. Creatinine 9.2–10.5 mg/dL, pre-dialysis BUN 47.5–53.7 mg/dL and post-dialysis BUN 15.4–18.9 mg/dL were within acceptable clinical guidelines.

**Table 3:** Plasma lipid concentrations.

Time	Ramadan (TR)	1 month post-Ramadan (T1)	2 months post-Ramadan (T2)
TAG (mg/dL)	143 ± 104 (34)	139 ± 90 (38)^a^	162 ± 106 (37)^a^
TC (mg/dL)	169 ± 55 (35)^a^	173 ± 40 (38)^b^	184 ± 48 (38)^ab^
HDL-C (mg/dL)	36 ± 10 (35)^ab^	30 ± 10 (38)^ac^	40 ± 14 (38)^bc^
LDL-C (mg/dL)	104 ± 45 (34)^a^	115 ± 32 (38)	114 ± 39 (37)^a^
TC/HDL-C	5.24 ± 2.70 (35)^a^	6.30 ± 2.48 (38)^ab^	5.29 ± 2.51 (38)^b^
LDL-C/HDL-C	3.24 ± 1.98 (34)^a^	4.22 ± 1.84 (38)^ab^	3.30 ± 1.79 (37)^b^
TAG/HDL-C	4.84 ± 4.61 (34)	5.38 ± 4.66 (38)	5.29 ± 5.09 (37)

Values are mean ± SD for the numbers in parentheses. Abbreviations: TR: during Ramadan; TC: total cholesterol; HDL-C: high-density lipoprotein cholesterol; TAG: triglyceride; Calc. LDL-C: calculated low-density lipoprotein cholesterol. ^abc^ Values in a given row sharing a common superscript were significantly different *p* < 0.05.

**Table 4:** Cholesterol distribution in lipoprotein subfractions.

Time	Ramadan (TR)	1 month post-Ramadan (T1)	2 months post-Ramadan (T2)
Large HDL (mg/dL)	11.2 ± 7.1 (35)^a^	10.1 ± 7.0 (38)^b^	13.8 ± 9.7 (38)^ab^
Intermediate HDL (mg/dL)	19.7 ± 4.4 (35)^ab^	16.0 ± 4.6 (38)^ac^	21.3 ± 6.7 (38)^bc^
Small HDL (mg/dL)	4.2 ± 2.0 (35)^a^	4.0 ± 2.2 (38)^b^	4.9 ± 2.4 (38)^ab^
Large LDL (mg/dL)	21.0 ± 13.3 (35)	18.3 ± 8.7 (38)^a^	23.5 ± 13.0 (37)^a^
Intermediate LDL (mg/dL)	9.0 ± 8.5 (35)^ab^	12.9 ± 9.0 (38)^a^	13.6 ± 10.0 (37)^b^
Small LDL (mg/dL)	1.8 ± 3.0 (35)^ab^	6.0 ± 9.3 (38)^a^	5.3 ± 9.0 (37)^b^
Mean LDL size (Å)	272.4 ± 4.2 (35)^ab^	268.2 ± 6.7 (38)^a^	268.9 ± 7.4 (37)^b^

Values are mean ± SD for the numbers in parentheses. Abbreviations: HDL-C: high-density lipoprotein cholesterol; LDL-C: low-density lipoprotein cholesterol. ^abc^ Values in a given row with common superscripts were significant at *p* < 0.05.

**Table 5:** Anthropometric assessment and malnutrition-inflammation scores.

Time	Pre-Ramadan (T-1)	Ramadan (TR)	1 month post-Ramadan (T1)	2 months post-Ramadan (T2)
BMI	24.0 ± 6.16 (35)	23.9 ± 6.42 (35)	23.9 ± 6.33 (35)	23.8 ± 6.36 (35)
Pre-D Wt. (kg)	63.5 ± 17 (36)	63.1 ± 17 (36)	63.3 ± 18 (36)	63.0 ± 17 (36)
Post-D Wt.	61.1 ± 17 (36)	60.7 ± 17 (36)	60.8 ± 17 (36)	60.6 ± 17 (36)
HGS (kg)	No data	19 ± 7.1 (33)	19 ± 6.8 (32)	19.0 ± 7.9 (33)
Mean TSF (mm)	No data	16.8 ± 10.8 (19)^ab^	20.5 ± 9.9 (19)^a^	21.1 ± 10.1 (19)^b^
MAMC (cm)	No data	21.4 ± 5.6 (19)	22 ± 5.1 (19)	22.2 ± 5.2 (19)
cAMA	No data	30.8 ± 22 (19)	32.8 ± 22 (19)	33.3 ± 22 (19)
MIS total scores^£^	No data	11 ± 4 (34)^ab^	10 ± 4 (33)^ac^	10 ± 4 (34)^bc^

Values are mean ± SD for the number in parentheses. Abbreviations: BMI: body mass index; Pre-D Wt.: pre-dialysis weight; HGS: hand grip strength; MIS: malnutrition-inflammation score; TSF: triceps skinfold; MAMC: mid-arm muscle circumference; cAMA: corrected arm muscle area. ^£^ Total score = sum of 10 components (0–30). ^abc^ Values sharing a common superscript were significantly different from each other (*p* < 0.05).

**Table 6:** Diet assessment.

		All Subjects			Acceptable reporters		
		Ramadan (TR)	1 month post-Ramadan (T1)	2 months post-Ramadan (T2)	Ramadan (TR)	1 month post-Ramadan (T1)	2 months post-Ramadan (T2)
Energy	Total kcal	1805 ± 736(30)^ab^	1256 ± 287 (29)^a^	1284 ± 421 (29)^b^	2139 ± 709 (24)^ab^	1767 ± 331 (21)^a^	1755 ± 424 (21)^b^
	kcal/kg IBW	32 ± 14 (30)^ab^	22 ± 7.6 (29)^a^	23 ± 8.9 (29)^b^	33 ± 12 (24)^ab^	27 ± 6 (21)^a^	27 ± 5 (21)^b^
Protein	Total g	60 ± 24 (30)^ab^	42 ± 10 (29)^a^	45 ± 15 (29)^b^	69 ± 24 (24)^ab^	56 ± 22 (21)^a^	60 ± 23 (21)^b^
	g/kg IBW	1.1 ± 0.5 (30)^ab^	0.7 ± 0.2 (29)^a^	0.8 ± 0.3 (29)^b^	1.1 ± 0.4 (24)^ab^	0.8 ± 0.3 (21)^a^	0.9 ± 0.03 (21)^b^
Carbohydrate	Total g	214 ± 86 (30)^ab^	170 ± 46 (29)^a^	165 ± 55 (29)^b^	248 ± 99 (24)	239 ± 73 (21)	221 ± 70 (21)
Fat	Total g	79 ± 41 (30)^ab^	48 ± 15 (29)^a^	52 ± 21 (29)^b^	97 ± 38 (24)^ab^	68 ± 24 (21)^a^	73 ± 25 (21)^b^
Potassium	Total mg	1405 ± 766 (30)^a^	1187 ± 753 (29)^b^	953 ± 434 (29)^ab^	1552 ± 846 (24)^a^	1495 ± 892 (21)	1088 ± 406 (21)^a^
Phosphate	mg/kg IBW	9.3 ± 4.6 (30)	9.05 ± 2.6 (21)	8.9 ± 4.0 (29)	10.5 ± 5.9 (24)	9.3 ± 6.2 (21)	9.7 ± 6.3 (21)

Values are mean ± SD for the number in parentheses. DEI: dietary energy intake; DPI: dietary protein intake; IBW: ideal body weight. Acceptable reporters with energy intake:total energy expenditure (EI:TEE) > 0.76. ^ab^ Values sharing a common superscript were significantly different from each other (*p* < 0.05).

## Supplementary Materials

The following are available online at https://www.mdpi.com/2072-6643/11/9/2225/s1, Table S1: Biochemical assessment.
