# Supplementary material for: Lipids, Lipoprotein Distribution and Nutritional Parameters over the Ramadan Period in Hemodialysis Patients
Source: Nutrients. 2019 Sep 14;11(9):2225. doi: 10.3390/nu11092225 (PMC6769954; doi:10.3390/nu11092225)
Supplement: Supplementary file 1 [file nutrients-11-02225-s001.pdf]

Table S1: Biochemical Assessment

Time	Pre-Ramadan (T-1)	Ramadan (TR)	1 month-post Ramadan (T1)	2 months-post Ramadan (T2)
Kt/V	1.34 ± 0.27(30)	1.34 ± 0.30(34)	1.36 ± 0.35(30)	1.38 ± 0.32(34)
Calcium (mg/dl)	8.7 ± 0.93(45)	8.7 ± 0.73(45)	8.8 ± 0.77(45)	8.9 ± 0.79(43)
Ferritin (ng/ml)	1206 ± 1036(39)	1221.9 ± 1199(40)	1101.4 ± 514(33)	1218 ± 1136(40)
Glucose - Random (mg/dl)	129 ± 63(42)	138 ± 90(42)	155 ± 135(41)[a]	127 ± 79 (42)[a]
Vitamin D (ng/ml)	24 ± 8.1(40)[a]	29.6 ± 9.5(41)[a]	No data available	26.3 ± 9.6(42)
Phosphorus (mg/dl)	5.5 ± 2.02(45)	6.0 ± 2.61(45)	5.9 ± 2.39(45)	5.8 ± 1.99(43)

Values are reported as mean± SD for the number in the parenthesis. Values in a given row, sharing a common superscript were significantly different from each other (p<0.05).
